# Hydrogen Sulfide Attenuates LPS-Induced Acute Kidney Injury by Inhibiting Inflammation and Oxidative Stress

**DOI:** 10.1155/2018/6717212

**Published:** 2018-01-31

**Authors:** Yuhong Chen, Sheng Jin, Xu Teng, Zhenjie Hu, Zhihong Zhang, Xuan Qiu, Danyang Tian, Yuming Wu

**Affiliations:** ^1^Department of Physiology, Hebei Medical University, Hebei, China; ^2^Intensive Care Unit, The Fourth Hospital of Hebei Medical University, Hebei, China; ^3^Department of Endocrinology, The Third Hospital of Hebei Medical University, Hebei, China; ^4^Hebei Collaborative Innovation Center for Cardio-Cerebrovascular Disease, Hebei, China; ^5^Key Laboratory of Vascular Medicine of Hebei Province, Hebei, China

## Abstract

In order to investigate the protective mechanism of hydrogen sulfide (H_2_S) in sepsis-associated acute kidney injury (SA-AKI), ten AKI patients and ten healthy controls were enrolled. In AKI patients, levels of creatinine (Cre), urea nitrogen (BUN), tumor necrosis factor-*α* (TNF-*α*) and interleukin-1*β* (IL-1*β*), and myeloperoxidase (MPO) activity as well as concentrations of malondialdehyde (MDA) and hydrogen peroxide (H_2_O_2_) were significantly increased compared with those of controls. However, plasma level of H_2_S decreased and was linearly correlated with levels of Cre and BUN. After that, an AKI mouse model by intraperitoneal lipopolysaccharide (LPS) injection was constructed for *in vivo* study. In AKI mice, H_2_S levels decreased with the decline of 3-MST activity and expression; similar changes were observed in other indicators mentioned above. However, the protein expressions of TLR4, NLRP3, and caspase-1 in mice kidney tissues were significantly increased 6 h after LPS injection. NaHS could improve renal function and kidney histopathological changes, attenuate LPS-induced inflammation and oxidative stress, and inhibit expressions of TLR4, NLRP3, and caspase-1. Our study demonstrated that endogenous H_2_S is involved in the pathogenesis of SA-AKI, and exogenous H_2_S exerts protective effects against LPS-induced AKI by inhibiting inflammation and oxidative stress via the TLR4/NLRP3 signaling pathway.

## 1. Introduction

AKI is a clinical syndrome caused by various factors and characterized by a rapid decline in renal function. Sepsis is the most common cause of AKI (40%–50%) in critically ill patients, and the mortality of SA-AKI is as high as 70% [1, 2]. The pathogenesis of SA-AKI is complicated, including inflammation, coagulation cascade activation, oxidative stress, microcirculatory disturbance, renal hypoperfusion, and renal venous congestion [3, 4]. Despite the growing understanding of the pathophysiological mechanisms of AKI, the development of pharmacological treatments for AKI shows little progress.

LPS is a normal component of cell wall of most Gram-negative bacteria, which can trigger cytokine synthesis, secretion, and a subsequent inflammatory process. Previous studies have demonstrated that LPS is one of the most important causes of sepsis and is involved in the pathogenesis of SA-AKI, which may lead to “cytokine storm,” intensified oxidative stress, low blood pressure, renal hypoperfusion, and finally a gradual decline in renal function [5–7]. Toll-like receptors (TLRs) are the most important pattern-recognition receptors and play a key role in innate immunity. The major receptor for LPS is TLR4 [8, 9], which is activated after LPS binding, and induces downstream signaling cascades as well as expression of inflammatory cytokines [10]. Nucleotide-binding domain and leucine-rich repeat protein-3 (NLRP3) inflammasome is one of the most important members of the Nod-like receptor (NLR) family, including NLRP3, the adaptor protein ASC, and a cysteine protease caspase-1. Abderrazak et al. [11] have found that NLRP3 inflammasome participated in the inflammatory process and may be critical for the development of sepsis and AKI. Activation of NLRP3 inflammasome could promote the maturation and release of proinflammatory cytokines IL-1*β* and IL-18. Another study found that NLRP3 expression induced by LPS was dose- and time-dependent, but it failed to induce NLRP3 expression in cells in the absence of TLR4 [12].

H_2_S was considered to be toxic for a long time until endogenous H_2_S was discovered in the rat brain [13]. H_2_S is mainly synthesized from L-cysteine by two pyridoxal-5′-phosphate- (PLP-) dependent enzymes, namely, cystathionine *β*-synthase (CBS) and cystathionine *γ*-lyase (CSE), and one phosphate-independent enzyme, 3-mercaptopyruvate sulfurtransferase (3-MST) [14]. Recently, a new H_2_S-synthesis pathway from D-cysteine by 3-MST along with D-amino acid oxidase (DAO) was discovered; moreover, D-cysteine was shown to be significantly superior to L-cysteine, particularly in the kidney and cerebellum [15]. Numerous studies have shown that H_2_S might have a potential therapeutic effect on ischemia/reperfusion injury (IRI) in multiple organs and metabolic diseases by inhibiting inflammation and oxidative stress [16–21]. Huang et al. [16] have demonstrated that H_2_S could protect against high glucose-induced inflammation and apoptosis by inhibiting the TLR4/NF-*κ*B pathway and NLRP3 activation in H9c2 cardiomyocytes. Zhang et al. [19] suggested that treatment with H_2_S could attenuate endotoxin-induced lung inflammation by inhibiting inducible nitric oxide synthase (iNOS) expression and nitric oxide (NO) production. Shibuya et al. [15] have reported that administration of D-cysteine could increase the H_2_S level in kidney tissues and protect renal function from IRI. Ahmad et al. [20] and Han et al. [21] also demonstrated that exogenous H_2_S accelerates kidney repair after IRI by inhibiting oxidative stress and inflammation. Although various effects of H_2_S in disease have been discovered, the role of H_2_S in the inflammatory process of sepsis has been a matter of debate for a long time. Whether H_2_S protects renal function by regulating inflammation and oxidative stress in SA-AKI remains unclear.

The purpose of the trial was to examine the effect of H_2_S on renal function during sepsis and the underlying mechanisms of H_2_S in SA-AKI.

## 2. Materials and Methods

### 2.1. Patients and Blood Sample Collection

Ten patients with AKI (AKI group) were recruited from the ICU at the Fourth Hospital of Hebei Medical University in China from June 2016 to November 2016, and ten healthy donors (control group) were selected from the age-matched population. AKI is defined as an abrupt decrease in renal function (within 48 h) and diagnosed according to the definition of the Acute Dialysis Quality Initiative group [22]. All patients with AKI were infected with Gram-negative bacteria (covering 2 cases from *Acinetobacter baumannii*, 2 cases from *Pseudomonas aeruginosa*, 2 cases from *Klebsiella*, 3 cases from *Escherichia coli*, and 1 case from *Clostridium difficile*), and renal injury caused by renal and postrenal factors has been excluded. None of the patients had been treated with drugs that cause kidney damage before. The patients who are younger than 18 years old, pregnant, or with a history of chronic renal dysfunction were excluded. The general condition and medical history of all participants were recorded. Blood samples were prospectively obtained from each participant at admission; after centrifugation for 10 minutes at 4000 rpm, the supernatants were frozen at −80°C. Each patient provided written informed consent prior to the study. The protocol was approved by the Ethics Committee of the Fourth Hospital of Hebei Medical University (Shijiazhuang, China) and performed in accordance with all applicable laws, regulations, and guidelines in China (including good clinical practice guidelines), which are relating protection of human subjects as volunteers. The study complied with the published regulations of the Declaration of Helsinki.

### 2.2. Drugs and Chemicals

LPS and NaHS were obtained from Sigma (Sigma–Aldrich, USA). NaHS was prepared 30 minutes before use. Detection kits for TNF-*α* and IL-1*β* levels were purchased from Xinbosheng Bioengineering (Shenzhen, China). Detection kits for MDA, H_2_O_2_, and MPO activities were obtained from Jiancheng BioEngineering (Nanjing, China). Dihydroethidium (DHE) was obtained from Beyotime Biotechnology (Shanghai, China) and, before use, dissolved in dimethyl sulfoxide (DMSO) to the desired concentration. Bicinchoninic acid (BCA) reagent was purchased from Generay Biotechnology (Shanghai, China).

### 2.3. Animals and Treatments

Male wild-type C57BL/6 J mice (8–10 weeks) were purchased from Vital River Laboratories (Beijing, China), subsequently housed and bred in standard conditions (12 h light/dark cycle) under constant temperature (22°C ± 2°C) and humidity (60%), and given free access to food and water.

After two weeks, 24 mice were divided into three groups randomly (*n* = 8): control, LPS, and LPS + NaHS (50 *μ*mol/L) groups. The LPS and LPS + NaHS groups were i.p. injected with LPS (5 mg/kg); in the control group, mice received an equal volume of saline (i.p.). Three hours after LPS treatment, the LPS + NaHS group received NaHS (50 *μ*mol/kg, i.p.); the LPS group were injected with an equal volume of saline (i.p.). The blood and kidney tissues of mice were collected 6 h after LPS treatment. Blood samples were immediately spun for 10 minutes at 4000 rpm and frozen at −80°C. The left kidney tissues were fixed with paraformaldehyde (4%), and the right kidney tissues were frozen at −80°C. All animal studies were carried out under the Guide for the Care and Use of Laboratory Animals (1985, NIH) after a review by the Ethics Committee for Laboratory Animals Care and Use of Hebei Medical University.

### 2.4. Histological Analysis

The kidney tissues were fixed with paraformaldehyde (4%), after 48 hours, paraffin-embedded and sectioned into 4 *μ*m thick, and then processed for hematoxylin and eosin (H&E) staining and evaluated using a light microscope (Olympus BX40, Tokyo, Japan). Kidney histological changes were scored as the previous study described [23] and evaluated in a double-blind fashion. Kidney damage was estimated at 5 randomly selected fields in the outer medulla, and tubular injury was defined as cellular degeneration and vacuolization, reduction of brush border epithelium, tubular obstruction, and cast formation. According to the following criteria: 0 = normal; 1 = damage less than 25% of tubular area; 2 = damage between 25% and 50% of tubular area; 3 = damage between 50% and 75% of tubular area; and 4 = damage between 75% and 100% of tubular area.

### 2.5. Measurements of Cre and BUN Levels in Plasma

Plasma Cre and BUN levels in patients and mice were measured using an automatic biochemical analyzer (Cobas 6000, Roche, Switzerland).

### 2.6. Measurement of Inflammatory Cytokines in Plasma

The enzyme-linked immunosorbent assay (ELISA) kits were used to determine the plasma TNF-*α* and IL-1*β* levels in patients and mice.

### 2.7. Measurement of the MPO Activity and MDA and H_2_O_2_ Concentrations

The plasma of patients and kidney tissues of mice were collected as described above. MPO activity and MDA and H_2_O_2_ concentrations were measured with colorimetric detection kits.

### 2.8. Measurement of Reactive Oxygen Species (ROS) Level

ROS level in kidney tissues was measured by staining the fresh frozen sections with DHE. Fresh kidney tissues were mounted using Tissue-Tek O.C.T. Blocks were sectioned into 5 *μ*m-thick slices, washed twice with prepared PBS, and incubated for 30 minutes with DHE (10 *μ*mol/L). The resulting color reaction was immediately measured with a fluorescence microscopy (Leica, Germany).

### 2.9. Measurement of CSE and CBS and 3-MST Activities

CSE and CBS and 3-MST activities in kidney tissues were measured according to the previous study described [24]. Homogenates from kidney tissues were prepared in ice-cold PBS; after centrifugation at 4°C at 12,000 rpm for 20 minutes, the supernatants were separated for measurement and protein concentrations were quantified by BCA assay. The cofactor pyridoxal-5′-phosphate (2 mmol/L) and enzyme substrate L-cysteine (10 mmol/L) were incorporated to the supernatant; after incubation for 30 minutes, the mixture was used to detect CSE and CBS activity. The *α*-ketoglutarate (2 mmol/L) and L-cysteine (10 mmol/L) were incorporated to the supernatant; after incubation for 30 minutes, the mixture was used to detect 3-MST activity. The activities of these H_2_S-generating enzymes were calculated by measuring H_2_S concentrations in the reaction system and the amount of H_2_S produced per microgram protein per hour.

### 2.10. Measurement of H_2_S Levels

H_2_S levels in the plasma and kidney tissues were detected according to previously described methods [25]. Homogenates from kidney tissues were prepared using cold Tris-HCl (100 mmol/L, pH 8.5); after centrifugation at 4°C at 12,000 rpm for 20 minutes, the supernatants were separated for measurement and protein concentrations were quantified by BCA assay. 30 *μ*L supernatant or plasma, 80 *μ*L monobromobimane (MBB, Sigma–Aldrich), and 10 *μ*L ammonia (0.1%) were mixed, by shaking at room temperature for 1 h, and 10 *μ*L formic acid (20%) was added to stop the reaction. Following a 10-minute centrifugation (15,000 rpm, 4°C), clear supernatants were frozen at −80°C for detection. H_2_S levels were determined using a curve formed with sodium sulfide (0–40 *μ*mol/L) standards. Plasma H_2_S level was expressed as *μ*mol/L, and the H_2_S level in kidney tissues was expressed as *μ*mol/g of protein.

### 2.11. Western Blot Analysis

Frozen right kidney specimens were dispersed mechanically in cold RIPA buffer. Proteins in the supernatants were extracted and quantified by BCA assay. After electrophoresis on 10% SDS-PAGE gels, the proteins were blotted to polyvinylidene fluoride (PVDF) membranes (Millipore-Upstate). After blocking in nonfat milk (5%) for 40minutes at room temperature, the membranes were incubated at 4°C for 12–18 hours with antibodies against CSE, CBS, 3-MST, TLR4, NLRP3, caspase-1, and *β*-actin (CSE and CBS were from Proteintech, USA; 3-MST and TLR4 were obtained from Abcam, USA; NLRP3 was obtained from Abnova, China; and *β*-actin was obtained from Irvine, USA). After washing three times with TBST, the membranes were incubated with secondary antibodies (Proteintech, USA) at room temperature for 2 h. Blots were detected with an enhanced chemiluminescence detection system (Sage Creation, Beijing, China). Band intensities were quantified using ImageJ software.

### 2.12. Statistical Analyses

All data analyses were performed using SPSS software version 21.0 (SPSS Inc., Chicago, IL, USA). Results were expressed as the mean ± S.E.M. For a comparison of more than two groups, a one-way analysis of variance (ANOVA), followed by the Student-Newman-Keuls test for multiple comparison, was applied. Comparisons between two groups were assessed by *t*-test. Statistical significance was defined as a *p* value of <0.05.

## 3. Results

### 3.1. H_2_S Levels Correlated with Cre and BUN Levels in Patients with SA-AKI

**Figure 1:**
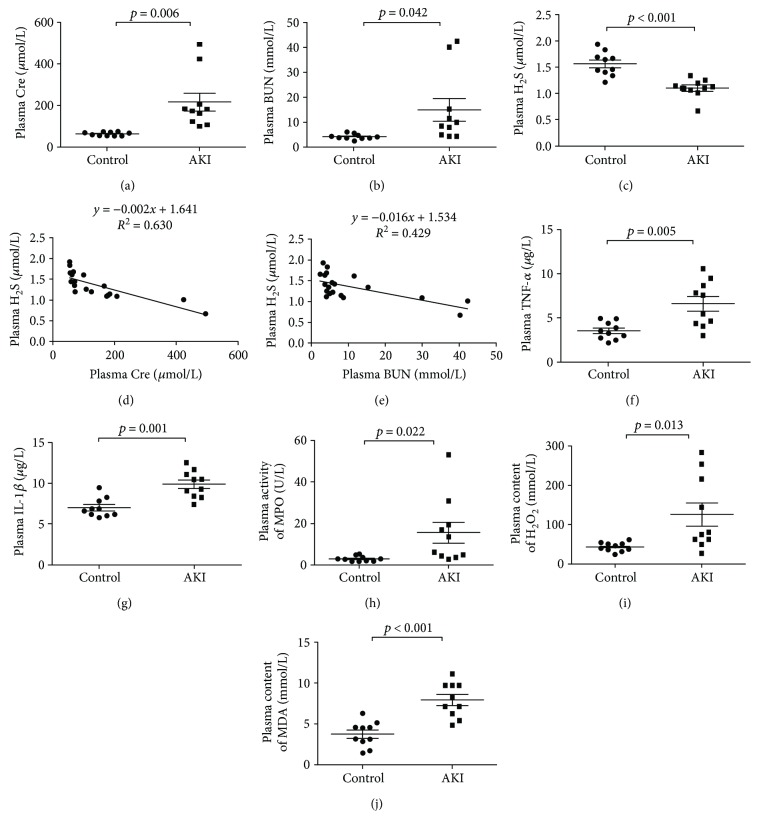
H_2_S levels decreased in patients with SA-AKI. (a) Cre levels in the plasma of control and SA-AKI patients. (b) BUN levels in the plasma of control and SA-AKI patients. (c) H_2_S levels in the plasma of control and SA-AKI patients. (d) Correlation between H_2_S and Cre. (e) Correlation between H_2_S and BUN. (f) TNF-*α* levels in the plasma of patients. (g) IL-1*β* levels in the plasma of patients. (h) The activities of MPO in the plasma of patients. (i) The concentrations of H_2_O_2_ in the plasma of patients. (j) The concentrations of MDA in the plasma of patients. Results are means ± SEM.

The characteristics of the 10 healthy donors and 10 AKI patients are shown in Table 1. There were no statistically significant differences between the two groups when comparing general features. Compared with controls, plasma Cre (Figure 1(a)) and BUN (Figure 1(b)) levels were significantly higher in patients with SA-AKI, whereas H_2_S levels were lower (Figure 1(c)). Furthermore, there was a significant inverse correlation between the levels of H_2_S and Cre (Figure 1(d)) or BUN (Figure 1(e)). This indicated that H_2_S level decreased during the pathological changes of SA-AKI.

### 3.2. Inflammatory Cytokine and Oxidative Stress Increased in Patients with SA-AKI

Plasma TNF-*α* and IL-1*β* levels increased in the AKI groups compared with those of the controls (Figures 1(f) and 1(g)). Furthermore, MPO activity and plasma H_2_O_2_ and MDA concentrations of patients were measured. The results were shown in Figures 1(h)–1(j). MPO activity and H_2_O_2_ and MDA concentrations increased in the SA-AKI group compared with those in the control group.

### 3.3. H_2_S Played a Role in LPS-Induced AKI

**Figure 2:**
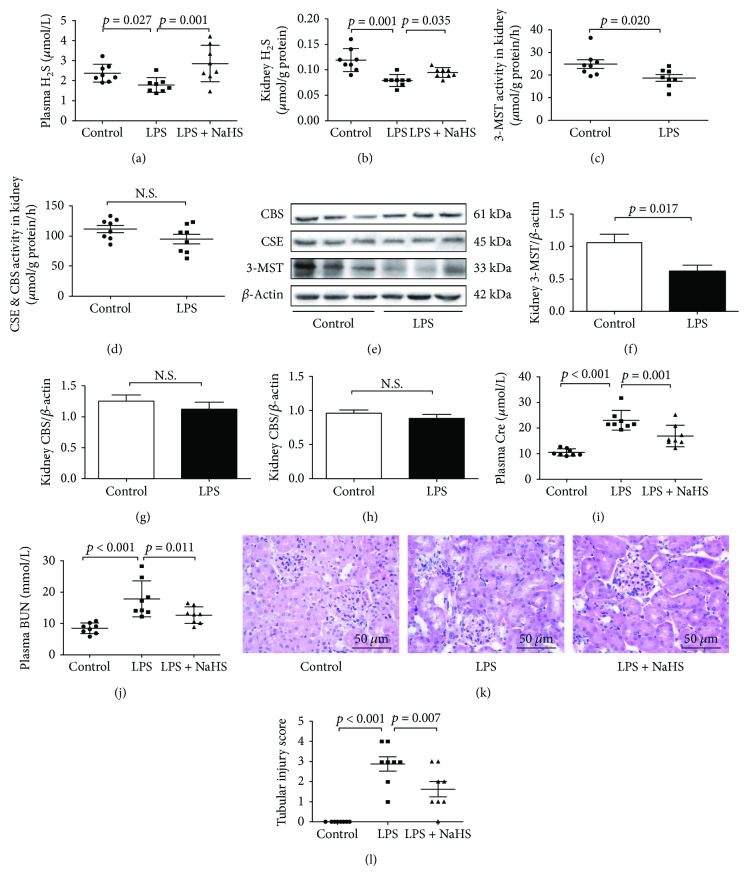
H_2_S levels decreased in mice with LPS-induced AKI, exogenous H_2_S could attenuate renal dysfunction. (a) H_2_S levels in plasma. (b) H_2_S levels in the kidney tissues. (c) 3-MST activity in the kidney tissues. (d) CBS&CSE activity in the kidney tissues. (e) Representative Western blots for 3-MST, CBS, and CSE expression in the kidney tissues. *β*-Actin was used as the internal control. (f−h) The quantitative analysis for 3-MST, CBS, and CSE expression in the kidney tissues. (i) Cre levels in plasma. (g) BUN levels in plasma. (k) Representative HE-stained right kidney sections (scale bar = 250 *μ*m). (l) Kidney tubular injury score of three groups.

In LPS-induced AKI mice, H_2_S levels in both the plasma and kidney tissues were significantly lower than those in the control groups (Figures 2(a) and 2(b)). H_2_S was generated by CBS, CSE, and 3-MST enzymes; therefore, the activity and expression of these three enzymes were measured in the kidney samples. 3-MST activity significantly decreased in the kidney tissues of LPS-induced AKI mice compared with that in controls, but there were no differences in CBS and CSE activities (Figures 2(c) and 2(d)). The expression of 3-MST significantly decreased in LPS-induced AKI mice compared with that in controls, whereas there were no differences in CBS and CSE expression (Figures 2(e)–2(h)). However, treatment with NaHS (50 *μ*mol/kg) significantly increased the LPS-induced reduction of H_2_S levels in the plasma and kidney tissues (Figures 2(a) and 2(b)), which was accompanied by a decline in plasma Cre and BUN levels (Figures 2(i) and 2(g)). In addition, NaHS treatment significantly ameliorated renal pathological changes caused by LPS. (Figures 2(k) and 2(l)).

### 3.4. Exogenous H_2_S Attenuated Inflammatory Cytokines and Oxidative Stress in Mice with LPS-Induced AKI

**Figure 3:**
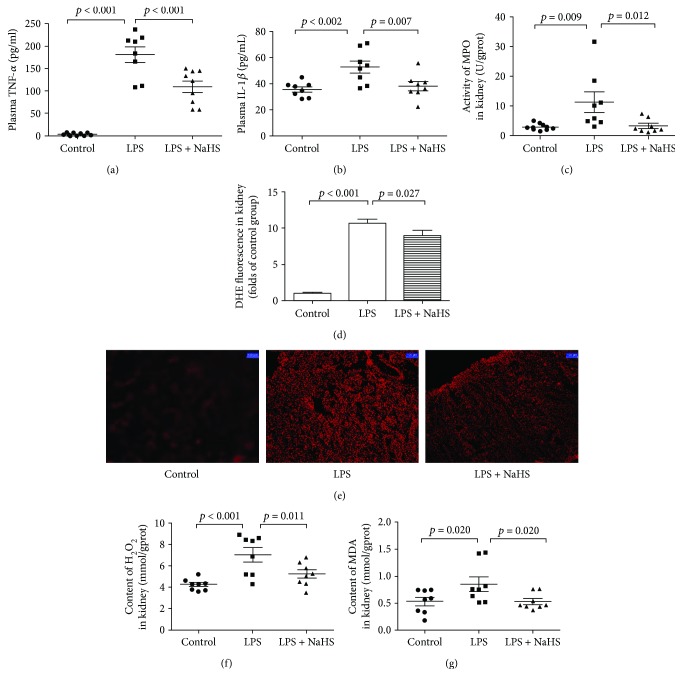
Exogenous H_2_S attenuated inflammatory cytokine and oxidative stress in mice with LPS-induced AKI. (a) TNF-*α* levels in the plasma of mice. (b) IL-1*β* levels in the plasma of mice. (c) The activity of MPO in the kidney tissue of mice. (d) The ROS levels in the kidney tissue of mice. (e) DHE fluorescence in the kidney tissue of mice. (f) The concentrations of H_2_O_2_ in the kidney tissue of mice. (g) The concentrations of MDA in the kidney tissue of mice. Results are means ± SEM.

Consistent with the performance in SA-AKI patients, plasma TNF-*α* and IL-1*β* levels, MPO activity, the ROS levels, and H_2_O_2_ and MDA concentrations in the kidney tissues significantly increased in mice with LPS-induced AKI. However, exogenous H_2_S could significantly attenuate these changes (Figures 3(a)–3(f)).

### 3.5. Exogenous H_2_S Attenuated the Formation of NLRP3 in the Kidney Tissues of Mice with LPS-Induced AKI

**Figure 4:**
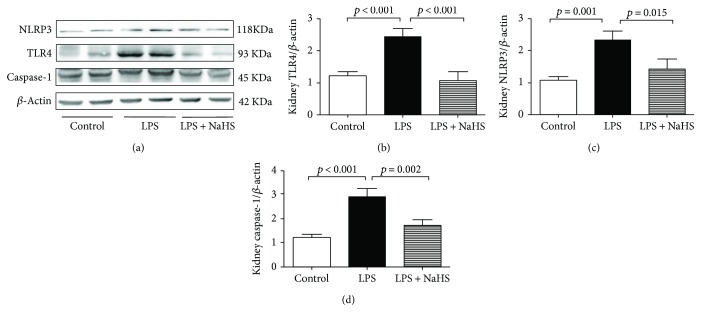
Exogenous H_2_S attenuated the formation of inflammasome in mice with LPS-induced AKI. (a) Representative Western blots for TLR4, NLRP3, and caspase-1 expression in the kidney tissues. *β*-Actin was used as the internal control. (b−d) The quantitative analysis for TLR4, NLRP3, and caspase-1 protein expression in the kidney tissues.

The exogenous effects of H_2_S on TLR4, NLRP3, and caspase-1 expressions were detected with Western blot analysis. The expressions of TLR4, NLRP3, and caspase-1 evidently increased in the LPS group compared with those of controls. However, exogenous H_2_S significantly decreased the protein expressions of TLR4, NLRP3, and caspase-1 (Figure 4).

## 4. Discussion

Numerous studies have shown the protective effects of H_2_S during diverse pathological processes [16–21]. Our study explored whether renal dysfunction during sepsis correlates with decreased H_2_S levels and other possible mechanisms. After assessing the results of this study, we found that (1) LPS decreased the level of endogenous H_2_S and reduced 3-MST activity and expression in the kidney and (2) exogenous supplement of NaHS ameliorated renal dysfunction by inhibiting inflammatory response and oxidative stress through TLR4/NLRP3 signaling pathways. These findings suggested that endogenous H_2_S contributes to the pathogenesis of SA-AKI, whereas exogenous H_2_S plays a protective role.

Sepsis is considered to be a systemic inflammatory response syndrome resulting from infection and often occurs in intensive care units. The related multiple organ dysfunction is a leading cause of death in critical patients. The kidney is the most vulnerable organ in sepsis, and mortality of SA-AKI may reach up to 70% [2]. The pathogenesis of SA-AKI is not completely understood. The three major changes that have been recognized in this disease process are inflammation, microcirculatory dysfunction, and cellular metabolic responses to sepsis [26]. LPS induces the release of inflammatory cytokines (especially TNF-*α* and IL-1*β*) through TLR4 signaling pathways and contributes to oxidative stress, which can activate tubular epithelial cells and result in functional damage to these cells, subsequently causing renal microcirculatory disturbance and hypoperfusion, which finally leads to SA-AKI [26–28]. The diagnosis of AKI mainly depends on clinical manifestations, urine volume, and blood biochemical markers. Cre and BUN are traditional and critical markers of renal function. MPO is a marker of neutrophil activity, while H_2_O_2_ and MDA are common markers of oxidative stress. In this study, we found that Cre and BUN levels, TNF-*α* and IL-1*β* levels, MPO activity, and H_2_O_2_ and MDA concentrations all significantly increased in the plasma of patients with SA-AKI. The mouse model of LPS-induced AKI was used to simulate the pathogenetic process of AKI, and similar changes were observed in the indicators mentioned above. These data were in agreement with the results of previous studies [6, 7, 10].

As was indicated in various studies, H_2_S played an essential role in the renal system, in both physiological and pathological status, and H_2_S levels were decreased in various kidney diseases [15, 20, 21]. To date, three enzymes and four pathways that are involved in the production of H_2_S have been reported. Kimura's team [14, 29] has demonstrated the formation of H_2_S from L-cysteine by CBS, CSE, and 3-MST coupled with cysteine aminotransferase (CAT); in addition, 3-MST along with DAO can produce H_2_S from D-cysteine. Furthermore, they found that the three enzymes are abundant in the kidney, but CBS and CSE are localized in the cytoplasm, whereas 3-MST is mainly found in the mitochondria, and the pathway of H_2_S production from D-cysteine by 3-MST/DAO only exists in the kidney and brain. To explain the effect of endogenous H_2_S in the course of SA-AKI, we measured the levels of H_2_S both in patients and mice with SA-AKI; similar to that demonstrated previously [15, 20], H_2_S levels significantly decreased and were inversely related to the renal function. To explore the reasons for the decline in H_2_S levels, we measured the activity of the three enzymes and their expression in the kidney tissues of mice and found that the activity and expression of both CBS and CSE in the kidney did not significantly change after LPS stimulation, but the 3-MST activity significantly decreased; moreover, 3-MST expression was downregulated, which may have contributed to the decrease of H_2_S. Although experimental studies [20, 21] indicated that protein expressions of CBS and CSE are decreased in kidney dysfunction caused by IRI models, the pathogenesis of SA-AKI is not exactly the same as that of ischemia reperfusion. From Kimura's report [29], we have also found that kidney D-cysteine can produce 60 times more H_2_S compared with L-cysteine, and D-cysteine is more efficient in protecting renal function. Therefore, we inferred that LPS may affect the H_2_S production mainly through the D-cysteine/DAO/3-MST pathway, although the mechanisms are still unclear and need further study.

On the basis of the above findings, we evaluated the potential therapeutic utility of exogenous H_2_S on SA-AKI. NaHS, an H_2_S donor, was extensively used in various animal models of renal IRI and proven to ameliorate kidney damage [21, 30]. Based on previous studies and preexperimental results, we administered NaHS (50 *μ*mol/kg) 3 h after LPS injection. NaHS had been demonstrated to increase H_2_S levels in both plasma and kidney tissues of LPS-induced AKI mice and H_2_S levels close to the normal range. Renal function and histological changes were also improved after NaHS administration, shown by a reduction of Cre and BUN levels in plasma as well as kidney injury score. These results were similar to those of Chen et al. [31], who found that the plasma H_2_S level is lower in rabbit with SA-AKI derived from the urinary tract, and treatment of NaHS could improve the H_2_S level, renal function, and pathological changes.

Inflammatory response is a cornerstone in the pathogenesis of SA-AKI. As one of the most important pattern-recognition proteins, TLR4 acts as a major receptor for LPS. Cunningham et al. [8] found that LPS binds to TLR4 in the kidney and induces the release of proinflammatory cytokines, especially TNF-*α* and IL-1*β*, which play an essential role during AKI. Fu et al. [32] demonstrated that inhibition of TLR4 could alleviate LPS-induced kidney injury. Meanwhile, Luo et al. [33] have demonstrated that NLRP3 is another important inflammatory regulator in sepsis-induced organ injury. NLRP3 could activate caspase-1, and subsequently, the cleavage and secretion of proinflammatory cytokines (IL-1*β* and IL-18) are promoted. Alfonso-Loeches et al. [34] showed the crosstalk between TLR4 and NLRP3 and found that activation of NLRP3 and secretion of inflammatory cytokines mostly disappeared in chronic alcohol-fed TLR4 knockout mice, which suggested that NLRP3 activation depends on TLR4 function. Over the years, various studies have shown the key role of H_2_S as a mediator of inflammation in various clinical settings. Shibuya et al. [15] demonstrated that H_2_S suppressed high glucose-induced cardiomyocyte inflammation by inhibiting the TLR4/NF-*κ*B pathway and NLRP3 activation. Moreover, Tan et al. [35] also identified the protective effect of endogenous H_2_S on renal IRI by suppressing inflammatory response through the inhibition of the TLR pathway. Zhang et al. [19] demonstrated that H_2_S could attenuate LPS-induced acute lung injury by reducing oxidative stress and inhibiting inflammation. Similar findings have been reported by Li et al. [36], who tested the role of H_2_S in mice with acute lung injury and found that exogenous H_2_S reduced lung permeability by suppressing oxidative stress and inflammation. Chen et al. [31] revealed that by inhibiting NF-*κ*B, H_2_S was able to decrease the plasma level of TNF-*α* and increase IL-10 level. To investigate the anti-inflammatory mechanism of H_2_S, we observed the effects of H_2_S on TLR4/NLRP3 signaling pathway and found that LPS could increase TLR4, NLRP3, and caspase-1 expression in kidney tissues, accompanied by plasma elevation of downstream inflammatory factors (TNF-*α* and IL-1*β*) and oxidative stress markers (MPO, H_2_O_2_, and MDA). In addition, our results showed that treatment with NaHS at 50 *μ*mol/kg could protect renal function by suppressing the release of inflammatory cytokines and oxidative stress, as well as TLR4, NLRP3, and caspase-1 expression.

## 5. Conclusion

In conclusion, our study suggested that endogenous H_2_S is involved in the pathogenesis of SA-AKI, and exogenous H_2_S exerts protective effects by inhibiting inflammation and oxidative stress via the TLR4/NLRP3 signaling pathway. These findings will shed light on the role of H_2_S as a therapeutic agent for renal diseases.

## Figures and Tables

**Table 1:** Characteristics of control and SA-AKI patients.

	Control (*n* = 10)	AKI (*n* = 10)	*p*
Age, yrs, mean [SD]	65.3 [8.6]	70.7 [16.0]	NS
Gender, male (%)	5 (50)	6 (60)	NS
BMI, kg/m^2^, mean [SD]	24.3 [1.8]	22.7 [2.2]	NS
Etiology of sepsis, by organ (*n*)	NA	Respiratory (4)	
		Abdominal (3)	
		Genitourinary (1)	
		Intestines (2)	
Previous history of chronic kidney disease (yes/no)	0/10	0/10	NS
WBC, ×10^9^/L, mean [SD]	5.4 [1.4]	14.3 [3.1]	<0.001
CRP, mg/dL	5.9 [1.5]	41.2 [25.5]	0.002

BMI: body mass index; WBC: white blood cell; CRP: C-reactive protein; NS = not significant; NA = not applicable.
